# Assessing the Statistical Significance of the Achieved Classification Error of Classifiers Constructed using Serum Peptide Profiles, and a Prescription for Random Sampling Repeated Studies for Massive High-Throughput Genomic and Proteomic Studies

**Published:** 2007-02-21

**Authors:** James Lyons-Weiler, Richard Pelikan, Herbert J Zeh, David C Whitcomb, David E Malehorn, William L Bigbee, Milos Hauskrecht

**Affiliations:** a Department of Pathology, Cancer Biomarkers Laboratory, Center for Pathology Informatics, Benedum Oncology Informatics Center; b Department of Computer Science; c Department of Surgery; d Departments of Medicine, Cell Biology & Physiology, and Human Genetics; e Clinical Proteomics Facility; f University of Pittsburgh Cancer Institute University of Pittsburgh

**Keywords:** ovarian cancer, pancreatic cancer, prostate cancer, biomarkers, bioinformatics, proteomics, disease prediction models, early detection

## Abstract

Peptide profiles generated using SELDI/MALDI time of flight mass spectrometry provide a promising source of patient-specific information with high potential impact on the early detection and classification of cancer and other diseases. The new profiling technology comes, however, with numerous challenges and concerns. Particularly important are concerns of reproducibility of classification results and their significance. In this work we describe a computational validation framework, called PACE (Permutation-Achieved Classification Error), that lets us assess, for a given classification model, the significance of the Achieved Classification Error (ACE) on the profile data. The framework compares the performance statistic of the classifier on true data samples and checks if these are consistent with the behavior of the classifier on the same data with randomly reassigned class labels. A statistically significant ACE increases our belief that a discriminative signal was found in the data. The advantage of PACE analysis is that it can be easily combined with any classification model and is relatively easy to interpret. PACE analysis does not protect researchers against confounding in the experimental design, or other sources of systematic or random error. We use PACE analysis to assess significance of classification results we have achieved on a number of published data sets. The results show that many of these datasets indeed possess a signal that leads to a statistically significant ACE.

## Introduction

High-throughput, low resolution time-of-flight mass spectrometry systems such as surface-enhanced laser desorption ionization - time of flight (SELDI-TOF) mass spectrometry (SELDI; Merchant and Weinberger, 2000; Issaq et al., 2002) and matrix-assisted laser desorption/ionization-time of flight mass spectrometry (MALDI) are just beginning to emerge as widely recognized high-throughput data sources for potential markers for the early detection of cancer (Wright et al., 1999; Adam et al., 2001; Petricoin et al., 2002). Spectra, or peptide profiles, are readily generated from easily collected samples such as serum, urine, lymph, and cell lysates. Comparisons have been made for a large number of cancers (Table 1) in search of diagnostic markers, with astonishingly good initial results for the classification of cancer and control profiles collected within respective studies.

With these very promising results the questions related to the significance and reproducibility of such classification results become imminent. Reproducibility and significance are essential with these types of data since the identity of the peptides located at clinically significant *m/z* positions that translate to the classification accuracy are unknown and their correctness cannot be verified through independent experimental studies.

The process of peptide profile generation is subject to many sources of systematic errors. If these are not properly understood they can potentially jeopardize the validity of the results. Such concerns have led to the analysis of possible biases present in published data sets and questions on the reproducibility of some of the obtained classification results under the proper experimental setup (Baggerly et al., 2004). Such studies highlight the need for randomization of sample order acquisition and processing, maintaining constant protocols over the course of a study (including sample handling and storage conditions), identification of potential confounding factors and the use of a balanced study design whenever possible to allow proper characterization of variation in the non-diseased population. Certainly, a design matrix should be created for each study and inspected for patterns that reflect complete or severe partial incidental confounding. In addition, multi-site validation studies, which are currently ongoing in the EDRN (Early Detection Research Network), can help to identify possible problems.

The peptide profile data are not perfect and include many random components. The presence of large amounts of randomness is a threat for interpretive data analysis; the randomness increases the possibility of identifying a structure and patterns in a completely uninformative signal. In such a case we want to have an additional assurance that the data and results of interpretive (classification) analysis obtained for these data are not due to chance. Permutation tests (Kendall, 1945; Good, 1994) used commonly in statistics offer one solution approach to this problem and allow us to determine the significance of the result under random permutation of target labels. In this work, building upon the permutation test theory, we propose a permutation-based framework called PACE (Permutation-Achieved Classification Error) that can assess the significance of the classification error for a given classification model with respect to the null hypothesis under which the error result is generated in response to random permutation of the class labels.

**Figure 1:**
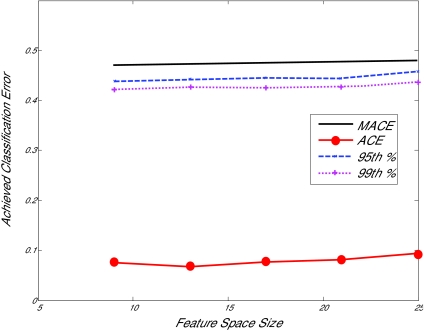
Example of PACE analysis. The permutation-achieved classification error (PACE) distribution is estimated by computing a statistic (in this case, testing error) over repeated relabeling of the sample data. The top solid line indicates the mean achieved classification error (MACE) of this distribution. The low 95^th^ and 99^th^ percentiles of the PACE distribution are given by the dashed and dotted lines, respectively. If the achieved classification error (ACE, bottom marked line) falls below a percentile band, it is a statistically significant result at that confidence level. In this example, ACE for a Naïve Bayes classifier using a weighted separability without peak selection or de-correlation (see below for details) falls consistently below the 99^th^ percentile band of the PACE distribution. It can be said that this classifier produced a statistically significant result at the 99% level.

The main advantage of the PACE analysis is that it is independent of the model design. This allows the problems of choosing the best disease prediction model and achieving a significant result to become decoupled. Many of the methods of high-throughput data analysis are very advanced, and thus may be poorly understood by the majority of researchers who would like to adopt a reliable analysis strategy. Understanding PACE analysis involves only visual examination of an intuitive graph (e.g, Figure 1), which makes it easy to apply and explain to the novice.

In the following we first describe the classification problem and evaluation of the classification performance. Next we introduce the PACE framework that offers additional assessment of the significance of the results. We compare PACE conceptually to existing confidence assessment methods; it is found to be potentially complementary to confidence interval-based bootstrap methods, which seek to determine whether a confidence interval around a statistic of interest includes a single point (or a series of single points; i.e, the ROC curve). Finally, we apply PACE analysis to a number of published and new SELDI-TOF-MS data sets. We demonstrate with positive and negative results the utility of reporting not only the ACE but also whether a given ACE is statistically significant. PACE thus provides a beginning reference point for researchers to determine objectively whether they have constructed a significant classifier in the discovery phase.

## Evaluation of classifiers

### Classification

Classification is the task of assigning class “labels” to sample data which come from more than one category. In our case, the classification task is to determine whether a particular proteomic profile comes from a case (cancerous) or control (non-cancerous) population. A classification model which assigns labels (either case or control) to profiles can be learned from training examples; profiles with known case and control labels. The goal is to achieve a classifier that performs as best as possible on future data. Practical concerns related to the classifier learning include the possibility of model overfit. The overfit occurs when the classification model is biased strongly towards training examples and generalizes poorly to new (unseen) examples. Typically, model overfit occurs due to the inclusion of too many parameters in the model in conjunction with a small number of examples. To assess the ability of the classification model to future data we can split the data from the study into *training and test sets*; the *training set* is used in the learning stage to build the classifier, the *testing set* is withheld from the learning stage and it is used for evaluation purposes only.

### Evaluation

*Training set:* a collection of samples used to identify features and classification rules based on discriminatory information derived from the comparisons of features between or among groups.

*Test set:* a collection of samples to which the classification rules learned from the training set are applied to produce an estimate of the external generalizability of the estimated classification error. The classification error rate observed when classifier is applied to them is called the test error rate. (Similarly, the sensitivity is called test set sensitivity, etc.). The classifier rules learned include parameters optimized using the training set that are then included in the prediction phase (for predictions on the test set). Test errors are usually higher than the training errors; Feng et al. refer to the difference as ‘optimism’; (Z. Feng, personal communication). Test errors are less biased than training errors, and therefore are more (but not completely) reflective of the expected classification error should the classifier be applied to new cases from the same population. The use of the test data set errors as the estimate is appropriate because it is low-biased compared to the classification errors achieved using only the training data set. The test set may be a held-out set of samples, or, more commonly, a number of held-out sets to avoid inaccuracy of ACE.

*Validation set:* a set of samples collected and/or processed and/or analyzed in a laboratory or at a site different from the laboratory or site where the original training sets were produced. Validation sets are never included in the learning step. All validation sets are test sets but not all test sets are validation sets. The more independence there is among sample sets, laboratory protocols, and implementation of a particular method of predicting class membership, the more robust the biomarkers.

### Cross-validation

Methods for estimating the test error include leave-one-out cross-validation, k-fold validation, and random subsampling validation. The selection of each of these depends in part on the number of samples available; these methods and their suitability for application to the analysis of high-throughput genomic and proteomic data sets have recently been explored (Braga-Neto & Dougherty, 2004). Use of the test error rates and performance measures derived from those rates allows one to assess the expected sensitivity (SN) and specificity (SP) of a given test or classifier; these performance measures are usually summarized in a confusion matrix. Even with these estimated performance measures, however, a more general question remains: for a broad range of potential outcomes and focus, from biomarker evaluation, discovery, validation and translation, what level of sensitivity is to be deemed significant, or sufficient, at a specified level of specificity? The clear overall objective of maximizing both SN and SP is built into the receiver-operator-characteristic (ROC) evaluation of a test, and the search of the most informative test usually seeks to maximize the area under the curve (AUC). Estimates of SN, SP, the ROC curve, and its area can all be determined using random subsampling validation. These approaches are well-studied, and their estimates of expected classification error are generally understood to be less biased than those estimated using training data sets.

## Permutation–based validation

The individual performance statistics by themselves, do not always allow us to judge the importance of the result. In particular, one should be always concerned by the possibility that the observed statistic is the result of chance. Careful elimination of this possibility gives more credibility to the result and establishes its potential importance. Permutation test methods offer a class of techniques that make this assessment possible under a wide variety of assumptions. Expected performance under the null model varies with the specifics of a design, and the distribution of the performance statistics vary with the distribution of information among markers and the type of disease prediction model used.

Permutation test methods work by comparing the statistic of interest with the distribution of the statistic obtained under the null (random) condition. Our priority in predictive models is to critically evaluate the observed discriminatory performance. In terms of hypothesis testing the null hypothesis we want to reject is:

The performance statistic of the disease prediction model on the true data is consistent with the performance of the model on the data with randomly assigned class labels.

The objective of optimizing a classification score itself is largely uncontrolled in most genomic and proteomic high-throughput analysis studies. Researchers do not, for example, typically attempt to determine and therefore do not report the statistical significance of the sensitivity of a test, in spite of the existence of a number of approaches for performing such assessments. Here we introduce a permutation method for assessing significance on the achieved classification error (ACE) of a constructed prediction model.

### Theory

A permutation test is a non-parametric approach to hypothesis testing, which is useful when the distribution for the statistic of interest *T* is unknown. By evaluating a classifier’s statistic of interest when presented with data having randomly permuted labels, an empirical distribution over *T* can be estimated. By calculating the *p*-value of the statistic’s value when the classifier is presented with the true data, we can determine if the classifier’s behavior is statistically significant with respect to the level of confidence α.

Let

$$\prod_{d}$$

be a set of all permutations of labels of the dataset with *d* examples. The permutation test (Mukherjee et al., 2003) is then defined as:

- Repeat *N* times (where *n* is an index from 1,…, *N*)
  - Choose a permutation
    $$\pi^{n}$$
    from a uniform distribution over
    $$\prod_{d}$$
  - Compute the statistic of interest for this permutation of labels
    $$t^{n}=T({\text{x}}_{1},y_{x_{1}^{n}},\ldots ,x_{d},y_{x_{d}^{n}})$$
    where
    $$x_{i},y_{\pi_{i}^{n}}$$
    denotes a profile-label pair, where the profile
    $$x_{i}$$
    is assigned the label according to the permutation
    $$\pi_{i}^{n}$$
- Construct an empirical cumulative distribution over the statistic of interest:
  $$\hat{P}(T\leq t)=\frac{1}{N}\left(\sum_{n-1}^{N}H(t-t^{n})\right)$$
  where H denotes the Heaviside function.
- Compute the statistic of interest for the actual labels,
  $$t_{0}=T({\text{x}}_{1},y_{1},\ldots ,{\text{x}}_{d},y_{d})$$
  and its corresponding p-value
  $$P_{0}=\hat{P}(T\leq t_{0})$$
  under the empirical distribution
  $$\hat{P}.$$
- If
  $$p_{0}\leq \alpha$$
  reject the null hypothesis.

For our purposes, the statistic of interest *T* is the achieved classification error (ACE).

### Application of permutation-based validation to peptide profiling (PACE)

We define a classification method *f* as all steps applied by a researcher to the data prior to some biological interpretation. These include the steps summarized in Table 2. In the case of SELDI/MALDITOF-MS, this may include mass calibration, baseline correction filtering, normalization, peak-selection, a variety feature selection and classification, approaches. We take the position that every researcher that has decided to approach the problem of analysis of a high-throughput proteomic data set has embarked on a journey of method development; i.e, the series of decisions made by the research itself is method *f.*

We assume that the researcher has adopted a study design that employs one or more training/test set splits, For our purposes, we use 40 random training/test splits to achieve a reasonably accurate estimate of ACE. A third validation sample can be set aside to verify the statistic on the pristine data. The validation set can either be produced at the same time, under the same conditions as the training/test data set. A more general estimate of the external validity of the estimate of the generalization error and its robustness to different laboratory conditions (and thus an assessment of the potential for practical (clinical) application) is obtained when the validation set is obtained at a different time or better yet in a different laboratory (as in multisite validation studies).

#### Permutation-Achieved Classification Error (PACE) Analysis

Given the achieved classification error (ACE) estimated via method *f*, generate an arbitrarily large number of new data sets with random sample relabeling. Method *f* is applied to each of the permuted data sets, resulting in a null distribution of ACE (called PACE). Lower 95th and 99th percentiles are located in PACE: ACE is then compared to these percentiles to assess the statistical significance of the classifier method *f*.

### Alternatives to PACE

The permutation-based approach compares the error achieved on the true data to errors on randomly labeled data. It tries to show that the result for the true data is different from results on the random data, and thus it is unlikely the consequence of a random process. We note that the permutation-based method is different and thus complementary to standard hypothesis testing methods that try to determine confidence intervals on estimates of the target statistics. We also note that one may apply standard hypothesis testing methods to check if the target statistic for our classification model is statistically significantly different from either the fully random, trivial or any other classification model. However, the permutation framework always looks at the combination of the data label generation and classification processes and thus establishes the difference in between the performance on the true and random data.

Classification error is a composite evaluation metric. Other types of performance measures for which confidence intervals have been studied so far include significance of SN at a fixed SP (Linnet, 1987), AUC (as implemented, for example, in Accu-ROC; Vida, 1993), and the ROC curve itself (Macskassy et al., 2003). Here we briefly explain these options. Which performance measure to assess may vary according to strategy. Bootstrap-estimated or analytically determined confidence intervals around SN at a specified SP (Linnet, 1987) requires that a desired SP be known, and this depends on its intent; for example a screening test should have very high SP to avoid resulting in too many false positives when applied to a population. Even here, however, “very high” and “too many” are rather context-dependent, should not be considered in a silo by ignoring existing or other proposed diagnostic tests. Acceptable FP values depend to a degree on the SP of existing practices, and to an extent on the prevalence of the disease. Any screen can be considered to change the prevalence of disease in the ‘potential patient’ population, and therefore follow-up with panels of minimally invasive markers, or multivariate studies of numerous risk factors (demographic, familial, vaccination, smoking history), and long-term monitoring, might make such screening worthwhile. High-throughput proteomics highlights the need for dynamic clinical diagnostics.

The various approaches suggested by Linnet were extended and revised with a suggestion by Platt et al. (2000) to adopt the bootstrap confidence interval method (Efron and Tibshirani, 1993). A working paper by Zhou and Qin (2003) explores related approaches. One strategy is to perform bootstrapping (Efron and Tibshirani, 1993) and calculate a 1-*α* confidence interval around a measure of interest. Bootstrapping is a subsampling scheme in which N data sets are created by sub-sampling the features of the original data set, with replacement. Each of the N data sets is analyzed. Confidence intervals around some measure of interest (*T*) can be calculated or consensus information can be gathered; in either case, variability in an estimate *T* is used a measure of robustness of *T.* Various implementations of the bootstrap are available; the least biased appears to be bias-corrected accelerated version (Efron and Tibshirani, 1993).

A second strategy is to calculate confidence intervals around the AUC measure. Bootstrapping (Efron and Tibshirani, 1997) is sometimes used to estimate AUC confidence intervals. Relying on confidence in the AUC can be problematic because it reports on the entire ROC, and, in practice, only part of the ROC is considered relevant for a particular application (e.g, high SP required by screening tests. A literature on assessing the significance of partial ROC curves has been developed (Dodd and Pepe, 2003; Gefen et al., 2003); a recent study (Stephan, Wesseling et al., 2003) compared the features and performance of eight programs for ROC analysis.

A third strategy is to calculate bootstrap confidence bands around the ROC *curve* itself (Macskassy et al., 2003). Under this approach, bootstrapping is explored and bands are created using any of a variety of ‘sweeping’ methods that explore the ROC curve in one (SN) or two (SN and 1-SP) dimensions.

## Experimental results of PACE analysis on clinical data

We applied PACE analysis to the following published data sets, and one new data set from the UPCI, using a number of methods of analysis:

- UPCI Pancreatic Cancer Data
- Ovarian Cancer Data (D1; Petricoin et al., 2002)
- Ovarian Cancer Data (D2; Petricoin et al., 2002)
- Prostate Cancer Data (Qu et al., 2002)

The *UPCI’s pancreatic cancer data* are only in the preliminary stages of analysis and we report only initial results. An ongoing study with an independent validation set is underway. Preoperative serum samples were taken from 32 pancreatic cancer cases (17 female, 15 male). Twenty-three non-cancer age, gender, and smoking history-matched controls were analyzed; ages ranged from 34 to 87, pancreatic cancer cases had a mean age of 64, controls had a mean age of 67 (p=0.19). Of the cancer samples, 16 were resected; 6 patients had locally advanced unresectable disease, and 10 had meta-static disease.

The *ovarian cancer datasets D1 and D2* (Petricoin et al., 2002) were obtained through the clinical proteomics program databank (http://ncifdaproteomics.com/). Both datasets were created from the same samples, but D2 was processed using a different chip surface (WCX2) as opposed to the hydrophobic H4 chip used to generate the data in D1. The samples consist of 100 controls: 61 samples without ovarian cysts, 30 samples with benign cysts smaller than 2.5 cm, 8 samples with benign cysts larger than 2.5 cm, and 1 sample with benign gynecological disease. The samples include 100 cases: 24 samples with stage I ovarian cancer, and 76 samples with stage II, II and IV ovarian cancer.

The *prostate cancer dataset* (Qu et al., 2002) was also acquired from the clinical proteomics program databank. It consists of 253 controls: 75 samples with a prostate-specific antigen (PSA) level less than 4 ng/ML, 137 samples with a PSA level between 4 and 10 ng/ML, 16 samples with a PSA level greater than 10 ng/ML, and 25 samples with no evidence of disease and PSA level less than 1 ng/ML. 69 cases exist in this dataset: 7 samples with stage I prostate cancer, 31 samples with stage II and III prostate cancer, and 31 samples with biopsy-proven prostate cancer and PSA level greater than 4 ng/ML.

### Methods Applied and Evaluated

Table 3 gives a summary of methods applied in the analysis. A brief description of some of these methods is provided below. A thorough description of these methods can be found in Hauskrecht et al. (2005, in press).

#### Peak detection

In some circles it is a strong belief that only peaks in a profile represent informative features of a profile. Peak detection can take place before performing further feature selection in order to limit the initial amount of the profile to be considered. There are various ways in which peak detection can be performed; for the purposes of our experiments, we utilize a peak detection method that examines the mean profile generated for all training samples, and then determines its local maxima. The local maxima positions become the only features considered for feature selection later in the pipeline displayed in Table 3. Alternatively, we can ignore the peak detection phase completely and consider the entire profile for feature selection.

#### Feature selection methods

##### Fisher Score

The Fisher score is intended to be a measure of the difference between distributions of a single variable. A particular feature’s Fisher score is computed by the following formula:

$$F(i)=\frac{{\left(\mu_{i}^{+}-\mu_{i}^{-}\right)}^{2}}{{\left(\sigma_{i}^{+}\right)}^{2}+{\left(\sigma_{i}^{-}\right)}^{2}}$$

where

$$\mu_{i}^{\pm }$$

is the mean value for the *i^th^* feature in the positive or negative profiles, and

$$\sigma_{i}^{\pm }$$

is the standard deviation. We utilize a variant of this criterion (Furey, 2000), computed with the following formula:

$$F(i)=\left|\frac{\mu_{i}^{+}-\mu_{i}^{-}}{\sigma_{i}^{+}+\sigma_{i}^{-}}\right|$$

To avoid confusion, we refer to the second formula above as our “Fisher-like score”. Features with high Fisher scores possess the desirable quality of having a large difference between means of case versus control groups, while maintaining low overall variability. These features are more likely to be consistently expressed differently between case and control samples, and therefore indicate good candidates for feature selection.

##### AUC Score (for feature selection)

Receiver operating characteristic curves are commonly used to measure the performance of diagnostic systems in terms of their “hit-or-miss” behavior. By computing the ROC curve for each feature individually, one can determine the ability of that feature to separate samples into the correct groups. Measuring the area under the ROC curve (Hanley et al., 1982) then gives an indication of the feature’s probability of being a successful biomarker. The AUC score for a given feature is then obtained by integrating over the ROC curve for that feature. As with the Fisher score, higher AUC scores signify better feature candidates.

##### Univariate *t*-test

The t-test (Baldi et al., 2001) can be used to determine if the case versus control distributions of a feature differ substantially within the training set population. The *t* statistic, representing a normalized distance measurement between populations, is given as

$$t_{i}=(\mu_{-}-\mu_{+})/\sqrt{\frac{\sigma_{-}^{2}}{n_{-}}+\frac{\sigma_{+}^{2}}{n_{+}}}$$

where

$$\mu_{-},\sigma_{-}$$

are the empirical mean and standard deviation for the *i^th^* feature in the

$$n_{-}$$

control samples, and

$$\mu_{+},\sigma_{+}$$

are likewise the empirical mean and standard deviation for the *i^th^* feature in the case samples. The *t* statistic follows a Student distribution with

$$f=\frac{{\left[(\sigma_{-}^{2}/n_{-})+(\sigma_{+}^{2}/n_{+})\right]}^{2}}{\frac{\left(\sigma_{-}^{2}/n_{-}\right)}{n_{-}-1}+\frac{\left(\sigma_{+}^{2}/n_{+}\right)}{n_{+}-1}}$$

degrees of freedom. For each feature, one can then calculate the *t* statistic and associated *f*, and determine the associated p-value with a predetermined confidence level from a standard table of significance. Smaller p-values indicate it is unlikely the observed case and control populations of the *i^th^* feature are similar by chance. Thus, it is likely that the *i^th^* feature is represented in a way that is statistically significant between case and control examples, making it a good candidate for feature selection.

We also evaluated feature selection using simple separability, weighted separability, and the J5 test (Patel and Lyons-Weiler, 2004).

##### De-correlation enhancement

After differential feature selection, we can perform further feature evaluation to avoid highly correlated features. These may be of interest for interpreting the biological sources of variation among peptides (such as carrier proteins; Mehta et al., 2003). For the purpose of constructing independent classifiers, however, it may be better to avoid using non-independent features - if only to increase the number of features included after feature selection - but also to avoid overtraining on a large number of highly correlated features. One way to avoid these correlated features is de-correlation (removal of features which are inter-correlated beyond some pre-determined threshold). All of the methods described so far can be evaluated with and without de-correlation.

##### Principal component analysis

Principal component analysis, a type of feature construction, incorporates aspects of de-correlation by grouping correlated features into aggregate features (components), which are presumed to be orthogonal (i.e, uncorrelated).

#### Classification models

##### Naïve Bayes

The Naïve Bayes classifier makes the assumption that the state of a feature (indicating membership in the case or control group) is independent of the states of other features when the sample’s class (case or control) is known. Let

$$X=\{x_{1},x_{2},\ldots ,x_{n}\}$$

be a sample consisting of *n* features, and

$$C=\{c_{1},c_{2},\ldots ,c_{m}\}$$

be a set of *m* target classes to which X might belong. One can compute the probability of a sample belonging to a particular class using Bayes’ rule:

$$p(c_{i}|X)=\frac{P(X|c_{i})P(c_{i})}{\sum_{j=1}^{m}P(X|c_{j})P(c_{j})}$$

The likelihood of sample *X* belonging to a particular target class *c_j_* is given as the product of each probability density function for each feature in the population of *c_j_*.

$$P(X|c_{j})\prod_{k-1}^{n}P(x_{k}|c_{j})$$

For our purposes, we assume each feature *x_k_* follows a Gaussian distribution, although other distributions are possible. Thus, the probability density function for feature *x_k_* is

$$P(x_{k}|c_{j})=\frac{1}{\sqrt{2\pi }\sigma_{kj}}\text{exp}\left(-\frac{1}{2}{\left(\frac{x_{k}-\mu_{kj}}{\sigma_{kj}}\right)}^{2}\right)$$

where

$$\mu_{kj},\sigma_{kj}$$

are the mean and standard deviation of the *k^th^* feature within the population of samples belonging to class *c_j_*. These two values, and their corresponding pair for the control population, must be estimated using the empirical information seen in the training set for each feature. The estimates are then used in the computation above during the predictive process on the testing set.

##### Support Vector Machine (SVM)

One might imagine a sample with *n* features as a point in an *n-*dimensional space. Ideally, we would like to separate the *n*-dimensional space into partitions that contain all samples from either case or control populations exclusively. The linear support vector machine or SVM (Vapnik 1995, Burges 1995) accomplishes this goal by separating the n-dimensional space into 2 partitions with a hyper-plane with the equation

$$w^{T}X+w_{0}=0$$

where *w* is the normal to the hyperplane, and

$$w_{0}$$

is the distance between the “support vectors”. These support vectors are the representative samples from each class which are most helpful for defining the decision boundary. The parameters of the model, **w** and

$$w_{0}$$

can be learned from data in the training set through quadratic optimization using a set of Lagrange parameters

$${\hat{\alpha }}_{i}$$

(Scholkopf 2002). These parameters allow us to redefine the decision boundary as

$${\hat{\text{w}}}^{T}\text{x}+w_{0}+\sum_{i\in SV}{\hat{\alpha }}_{i}y_{i}({\text{x}}_{i}^{T}\text{x})+w_{0}$$

where only samples in the support vector contribute to the computation of the decision boundary. Finally, the support vector machine determines a classification

$${\hat{y}}_{i}$$

for the *i^th^* sample as seen here:

$$\hat{y}=sign\left[\sum_{i\in SV}{\hat{\alpha }}_{i}y_{i}({\text{x}}_{i}^{T}\text{X})+w_{0}\right]$$

where negative

$${\hat{y}}_{i}\;s$$

will occur below the hyperplane, and positive

$${\hat{y}}_{i}\;s$$

will occur above it. Ideally, all samples from one group will have negative

$$\hat{y}$$

while all others will have positive

$${\hat{y}}_{i}$$

### PACE Results

All four cancer datasets were analyzed using classifiers defined by differing configurations of feature selection criteria, peak selection, de-correlation, and classification models. De-correlation MAC thresholds range from 1 (no de-correlation) to 0.4 (strict de-correlation) in increments of 0.2. To assess the statistical significance of the classifiers generated through these configurations, PACE analysis was performed using 100 random permutations of the data over 40 splits into training and testing sets. Classifiers were evaluated over the range of 5 to 25 features, in increments of 5 features.

For illustrative purposes, examples of PACE graphs are presented in the appendices of this work. These graphs represent only a portion of the classifiers evaluated for this work. In particular, the appendices present PACE graphs for SVM classifiers enforcing a 0.6 MAC threshold, both before and after peak selection, for each of the univariate feature selection methods.

### UPCI Pancreatic Cancer Data

**Figure A1:**
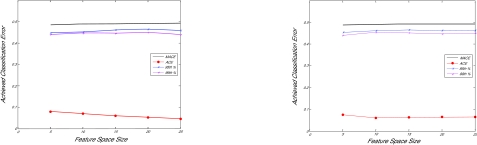
PACE analysis using a SVM-based classifier using the AUC scoring criterion, with a 0.6 MAC threshold. Left Panel: performance without peak selection. Right panel: performance with peak selection. In these figures, the upper solid line represents the Mean Achieved Classification Error (MACE) under the null hypothesis derived using sample class label permutations. The next two dotted lines represent the 95^th^ and 99^th^ percentile, and the lower solid line represents the ACE for the classifier (averaged over iterations).

Each possible configuration of classification models produced a statistically significant classifier at the 99% level. This trend was observed for all feature sizes in each classifier. See figures A.1 through A.6 for examples of PACE analysis on this dataset using different feature selection criteria.

#### Ovarian Cancer Data (D1; Petricoin et al., 2002)

**Figure B1:**
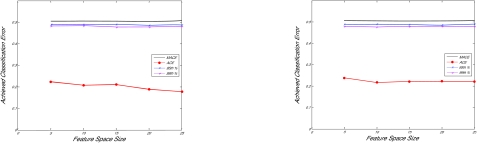
PACE analysis using a SVM-based classifier using the AUC scoring criterion, with a 0.6 MAC threshold. Left Panel: performance without peak selection. Right panel: performance with peak selection.

Each possible configuration of classification models produced a statistically significant classifier at the 99% level. This trend was observed for all feature sizes in each classifier. See figures B.1 through B.6 for examples of PACE analysis on this dataset using different feature selection criteria.

#### Ovarian Cancer Data (D2; Petricoin et al., 2002)

Each possible configuration of classification models produced a statistically significant classifier at the 99% level. This trend was observed for all feature sizes in each classifier.

**Figure C1:**
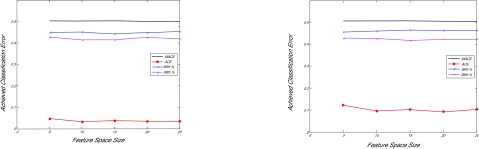
PACE analysis using a SVM-based classifier using the AUC scoring criterion, with a 0.6 MAC threshold. Left Panel: performance without peak selection. Right panel: performance with peak selection.

See figures C.1 through C.6 for examples of PACE analysis on this dataset using different feature selection criteria.

#### Prostate Cancer Data (Qu et al., 2002)

Under random feature selection, several classifiers were produced which were not statistically significant at the 99% or 95% level. Using the Naïve Bayes classification model, the generated classifiers were not significant at the 95% level for small amounts of features (5–15). As de-correlation becomes stricter, the classifiers lost statistical significance at high amounts of features where they had been significant with a more lenient MAC. When coupling this technique with peak selection, no statistically significant classifiers were produced. With an SVM-based classifier using random feature selection, the produced classifiers were significant at the 99% level except when using the initial 5 features. Changes in MAC and peak selection did not change this behavior.

In general, Naïve Bayesian classifiers using univariate feature selection criteria are significant at the 99% level as long as peak selection is not performed beforehand. The one exception was the J5 test, which was unable to produce a significant classifier at the 95% level without the aid of de-correlation. Applying de-correlation allowed these classifiers to achieve significance at the 99% level. When performing peak selection, only the classifiers produced using the strictest MAC thresholds (0.6, 0.4) were able to achieve some form of significance, and even then, only at high amounts of features (15–25). The weighted separability score was unable to produce a significant naïve Bayes classifier using peak selection.

SVM classifiers using univariate feature selection criteria were nearly always significant at the 99% level, either with or without peak selection. The few instances where there was no significance at the 95% level occurred using the J5 and simple separability scores without de-correlation. In the case of the J5 score, lowering the MAC to 0.8 remedied the situation, while the simple separability score improved simply through incorporating additional features.

**Figure D1:**
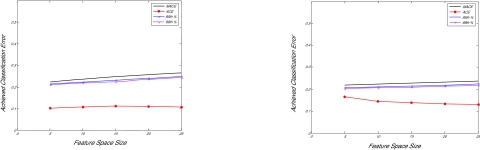
PACE analysis using a SVM-based classifier using the AUC scoring criterion, with a 0.6 MAC threshold. Left Panel: performance without peak selection. Right panel: performance with peak selection.

See figures D.1 through D.6 for examples of PACE analysis on this dataset using different feature selection criteria.

### Discussion

We have before us a daunting challenge of creating conduits of clear and meaningful communication and understanding between ‘consumers’ (statisticians, computational machine learning experts, bioinformaticians) and the producers of high throughput data sets. The objective is to maximize the rate at which clinically significant patterns can be discovered and validated. Disciplines can be bridged in part by a straightforward reference point on performance provided by decision-theoretic performance measures. Nevertheless, performance characteristics that are typically reported (SN, SP, PPV, NPV) only provide partial information on performance (the method’s performance in the alternative case). Researchers may be reluctant to publish results that have ‘relatively low’ SN and SP (e.g, 0.75, 0.8), and yet this level of performance may in fact be highly surprising given the sample numbers and degree of variability (due to noise variance). Stellar results such as high 90’s sensitivity and specificity predominate in the published cancer literature (Table 1), posing the question of whether the early reports of high performance may have set the standard too high. Some biological signal and powers of prognosis can be expected to be lower. Our work focuses on the question: what represents a remarkable SN? SP? AUC? ACE? We study this from the perspective that proteomic profiling represents only one of many different sources of potential clinically significant information, and that combined use of panels of biomarkers and other molecular and classical diagnostic information is likely to be required if proteomic profiling becomes widely adopted.

#### Minimize ACE: Conjecture or Tautology?

In microarray analysis, most papers describe a new algorithm or test for finding differentially expressed genes. This makes is difficult to assess the validity of a given analytical strategy (method of analysis). We recommend that a standard be considered for the assessment of the impact of particular decisions in the construction of an analytical strategy, including decisions made during pre-processing (Figs. 2 and 3): Specifically,

Any method that results in a significant ACE is to be preferred over methods that do not achieve significance. All significant methods (at a specified degree of significance) are equally justified – for the time being.

It is possible that different methods that achieve significant ACE will identify distinct feature sets, in which case each feature set is potentially interesting.

Note that we are not suggesting that reproducibility is not important; i.e, ideally, the same methods on similarly-sized different data sets should achieve similar levels of significance. Indeed, reproducibility is key; therefore, the methods that yield similar levels of significance in repeated experiences are also validated.

Note also that we are also *not* recommending that one should adopt the somewhat opposing position that

The method that minimizes ACE will tend to be most significant, and therefore will likely be best justified.

In contrast, we consider it likely that clinically significant information may exist at a variety of scales within these large data sets. The search for a method-any method- with the most significant ACE from a single data set seems likely to lead to overestimates of the expected clinical utility of a set of biomarkers. Comparisons of ACE across cancer types and with independent data set would be informative.

#### Nonsignificant Results

Reasons for negative results might include no biological signal, poor study design or laboratory SOPs, poor technology, or low biological signal (requiring larger numbers of samples). It is our position that researchers are better informed whether the result is significant or not. For example, a non-significant ACE may inform the researcher that they should refine or redirect their research question; an example might include early detection of a given disease providing a negative result in the pre-disease state, suggesting that one might move the focus to early stage disease instead of pre-disease. While the clinical prediction of a potential outcome during the course of disease may not be possible from the preconditioned state, the research might shift focus toward ‘how early can this condition be predicted?’ While we report few non-significant results, we have seen non-significant results from unpublished, proprietary studies of which we cannot report the details. The results are unpublished in part due to the negative results, and in part due to the changes in the experimental design that has resulted due to achieving a negative result.

#### Relation of PACE to Similar Methods

PACE creates a distribution of the expected ACE under the null condition. The fixed measure ACE is the average classification error over all random sub-samplings. This generates a distribution around ACE, and the determination of significance could involve a comparison of the degree of overlap between the ACE and PACE distributions. As we have seen, PACE is similar in focus to a number of alternative methods with slightly distinct implementations and foci. These include the ROC bootstrap confidence interval on AUC, confidence interval estimation around SN at a fixed SP, and bootstrap bands around the ROC curve itself. The bootstrap ROC is used to determine a confidence interval around an estimated area under the ROC curve (AUC); we are most interested in the specific part of feature space where a classifier works best, not in the overall performance of a classifier over a range of stringency, and thus PACE focuses on comparing a point estimate of statistic theta to its null distribution. A traditional limitation of permutation tests is an assumption of symmetry; in our case, we are only interested in the lower tail of the PACE distribution. In the case of individual performance measures (SN, SP) or the composite AUC, one would be interested only in the upper tail of ACE. Symmetry is also known to be an especially important assumption when estimating the confidence interval around the AUC (Efron and Tibshirani, 1993).

The question of relative suitability of these alternatives should be determined empirically to determine if any practical differences exist in this particular application. So the question is posed: which statistical assessment of confidence is of most practical (applied) interest: the specific measurement of classification error achieved by *x* in the learning stage of the actual study, or the distribution of the classification error in imagined alternative cases? We prefer to make our inferences on the data set at hand, for the time being, using imagined alternatives that involve a (hopefully) well-posed null condition. The bias-corrected accelerated bootstrap confidence interval (Efron and Tibshirani, 1993), which is range-respecting and range-preserving (and unbiased, as the name suggests) corrects for differences between the median AUC of some of the pseudosamples and that of the original sample, making the imagined alternative samples more like the actual sample. This method should also be explored in this context.

Some of these disparate methods could also potentially be combined (e.g, PACE as the null distribution and ROC bootstrapping to assess confidence intervals around ACE). This would use the degree of overlap of distributions instead of specific instance outside of a generated distribution. A more formal exploration of these possibilities seems warranted.

#### Robustness of PACE and Permutation Approaches to the Stark Realities of High-Throughput Science

PACE provide a reference point that is robust to many of the vagaries in study design common to peptide profiling studies, such as different numbers of technical replicates per sample that result from the application of QA/QC. Compared to distribution-dependent criteria that would otherwise require adjustments to degrees of freedom, both PACE and the bootstrap are relevant for the data set at hand.

#### Caveats

PACE and the other methods cited here do not protect incidental partial or complete confounding. True validation of the results of any high-throughput analysis should involve more than one site, ideally with the application of a specific classifier rule learned at site A to data generated at site B. Further, to protect against amplification of local biases by data preprocessing steps, the preprocessing must be wrapped within the permutation loop.

#### A Word on Coverage

It is important to consider in the development and evaluation of biomarker-based classification rule whether a sample is classifiable; i.e, do the rules developed and data at hand provide sufficiently precise information on a given sample. The proportion of samples that are predictable in a data is defined as *coverage.* If a strategy is adopted whereby a number of samples are not classified, the evaluation scheme (whether it be a bootstrap, random subsampling-derived confidence boundaries, or permutation significance test) should also be forced to not classify the same number of samples. These ‘enforced passes’ on a sample must be checked and enforced after the prediction stage to conserve the numerical and statistical aspects of the study design and data set (e.g, s, number of samples; variability within *m/z* class).

Research is needed to determine the importance of asymptotic properties, dependencies of the bootstrap ROC on the monotonic or jaggedness of the ROC curve, and the use of combined distributions (i.e, measure of degree of overlap between the PACE distribution, as the null distribution, and the bootstrap ROC curve as variability in the estimated classifier performance measure of interest in separate instance of the study).

#### Towards a More Complete Characterization of the Problem

In the consideration of further development and improvements in analytic methods for the analysis of peptide profiles, we assume that detailed descriptions of fundamental characteristics of low-resolution peptide profiles can be used to help set priorities in the construction of particular strategies. These descriptions/observations include

- an acknowledgement of somewhat high mass accuracy (0.2–0.4%);
- a comprehension that individual *m/z* values are not specific (i.e, they are not unique to individual peptides), and therefore intensity measures at a given *m/z* value reflect sum intensity of ‘peptide *m/z* classes’, which may or may not be functionally associated;
- an understanding that peptides do not map to single individual peptides; i.e, they exist two or more times in the profile at different *m/z* values as variously protonated forms. Each peptide may have a roughly unique signature, and pattern matching forms the basis of peptide finger-print data mining, but a peptide need not occur as a single peak;
- an understanding that *m/z* variance will contain biological sources (mass shifts due to amino acid sequence variation and varying degrees of ubiquination and cleavage, binding of peptides with others), chemical, and physical components (mass drift), and thus models that allow the statistical accounting of each of these variance components are needed;
- an understanding that high - intensity measurement in SELDI-TOF-MS profiles tend to exhibit higher variance, which suggest that reliance of peaks for any inference (analyzing peaks only, aligning peaks, or normalizing profiles to peaks) may add large, unwanted components of variance or restrict finding to peptides with intensities that are most inaccurately measured;
- the acknowledgment that the *m/z* vector is an arbitrary vector along which intensity values of similarly massed and charged peptides are arranged, and, as an arbitrary index in and of itself may require (or deserve) no profound biological explanation and may or may not offer a profound biological insight related to the clinical questions at hand beyond a guide to identity of peptide by pattern matching;
- observations that features determined to be significant tend to be locally correlated and that long-range correlations also exist, and that both artifactual and biologically important correlations and anti-correlations may exist at both distances;
- an expectation that correlations may exist that reflect protonated forms of peptides and that some correlation/anticorrelation pairs may reflect real peptide biology, such as enzymatic cleavage cascades;
- similarly, the observation that at least part of the local autocorrelation observed in the profile is likely due to poor resolution (mass drift), and reflects a physical property of the profiles (instrument measurement error and resolving power). It may also reflect smoothing due to natural biological variation in the population from which the samples were drawn, the effects of summing intensities of distinct peptides that share similar but not identical *m/z* values. One might consider whether the local correlations all reflect real biological properties of single peptides at particular *m/z* positions, and, if not, they may offer no biological insight and may require no biological explanation (i.e, local autocorrelation may be simple artifact of degree of resolution of the instrument and the lack of specificity of *m/z* values).

These descriptions may help motivate research on variance corrections, de-correlation, the use of PCA, profile alignment strategies, and attempts at transformation.

#### Other Open Questions

As high-throughput genomic and proteomic data become less expensive, and the laboratory equipment spreads into an increasing number of facilities, it seems likely that different laboratories will study the sample problem with completely independent effort. Published data sets, therefore, represent profoundly useful potential source of corroboration, or validation, of biomarker sets that might be expected to exhibit reproducible differences in large portions of the patient population. A careful characterization and validation of those differences, as a step that is independent of the question of potential clinical utility, is essential in these studies. True validation by planned repeated experiments may seem daunting, or unwarranted at this early stage, and the tendency will be to attempt to validate markers deemed to be significant in a small study using other technology (immunohistochemistry, for example). In this case, absence of validation of specific proteins with other technology is not complete refutation due to the potential for idiosyncrasies in this new application of mass spec technology. Computational validation applied at the step of feature selection alone could prove invaluable (i.e, which features are reproducibly different between cases and controls, responders and nonresponders, in independently analyzed subsets or splits of the data samples?)

#### Large multi-year and multi-site studies

As unlikely as large-scale repeated studies may seem, it seems imminent that studies of peptide profiles from thousands of patients and normal donors will be forthcoming. What are the practical problems in such a setting? We would advocate avoiding the temptation to view one large data set (say, 5,000 patient, 5,000 normal) as a single study, and would recommend analysis of multiple, random independent (non-overlapping) subsets, which would provide true validation of feature selection methods and classification inferences. Such large studies will occur over long timer periods. Laboratory conditions change, and manufacturers change kits and protocols; thus, to maximize the generalizability of the performance characteristics of a trained classifier, training and test sets should be randomly selected and blinded. We must remember that learning is asymptotic. Therefore, researchers should avoid evaluating a classifier built on training data set 1 produced at time 1 with testing set produced at time 2; instead, they should randomize the data over the entire time period, even if this means re-learning a classifier after publishing an initially internally valid classifier using data set 1. This approach still involves training, but protects against a biased (overly pessimistic) result due to shifts in laboratory conditions.

#### Future Directions in Peptide Profiling

Given that the distribution of pure noise variance over the *m/z* range is not uniform under the null condition, univariate feature selection methods such as t-tests, Fisher’s score, area under the curve (AUC) and their nonparametric alternatives are perhaps best applied as permutation tests to attempt to equalize the Type 1 error rate over the *m/z* range included in an analysis. When combined with PACE, this greatly increases the computational burden of analyzing even a small set of profiles, but the pay-off should be immense. Features that are not significant under the parametric, distribution-dependent tests can become significant under the permutation test for significance, and the reverse shifts are also possible. This becomes especially important when using significance levels to select *n*-ranked features. When permutation feature selection methods are then combined with classification algorithms such as PCA, SVM, or nearest neighbor algorithms, and then are evaluated by PACE or bootstrap methods, this clearly will require a large network dedicated to cancer proteomic analysis, and a consortium of developers dedicated to bringing well-known existing and new algorithms for analysis to bear on the important problems in cancer research, including early detection, recurrence, progression and therapy outcome. A plan to use the rational unified process outlined in caCORE (Covitz et al., 2003) as a software development protocol will help combine the energies of participants and developers in the Integrative Cancer Workspace of NCI’s caBIG workspace with those of participants in the EDRN is under development. We intend to build a parallel-processing friendly analysis framework so researchers can objectively evaluate and report the effects of the decisions they make during each stage in analysis. Even so, support of analysis for small (pilot) studies is needed, and we can reasonably expect that optimal analysis solutions to vary with study design. The reanalysis of published data sets will also be key to sorting through the method space, so the design of such a solution might include data sets ‘on-tap’, as we have done for caGEDA (http://bioinformatics.upmc.edu/GE2/GEDA.html) for microarray data analysis (Patel and LyonsWeiler, 2004). Simulations will also be key. We encourage sites to make their raw data (unpreprocessed) and source code available under an open source license to resolve analysis challenges as rapidly and as directly as possible.

## Figures and Tables

**Table 1:** Published sensitivities and specificities of SELDI-TOF-MS profiling for various types of cancers

Cancer Type	SN, SP	Reference
Ovarian Cancer	100%, 95%	Petricoin et al., 2002
Prostate Cancer	100%, 100%	Qu et al., 2002
Breast Cancer	90%, 93%	Vlahou et al., 2003
Breast Cancer	91%, 93%	Li et al., 2002
Head & Neck Cancer	83%, 90%	Wadsworth et al., 2004
Lung Cancer	93.3%, 96.7%	Xiao et al., 2003
Pancreatic Cancer	78%, 97%	Koopmann et al., 2004

**Table 2:** Steps in the Analysis of High-Throughput Peptide Profiling Spectra. These steps were elucidated in part in discussion with the EDRN Bioinformatics Working Group. We gratefully acknowledge their input.

Experimental Design	Selection of type and numbers of samples to compare
**Measurement**	Determination of sample rate Mass calibration
**Preprocessing**	Profile QA/QC filtering Variance correction/regularization Smoothing Baseline correction Normalization (internal or external)
**Data Representation**	Determination of profile attributes: Peak selection Whole-profile Partial-profile Binning May also include peak-finding algorithms and peak-matching routines
**Feature Selection**	Identification of profile features which are likely to be clinically significant: Univariate statistical analysis Multivariate feature selection
**Classification**	Rendering sample class inferences
**Computational Validation / Study Design**	Calculation of an estimated classification error rate which is hopefully unbiased and accurate. May involve: Random subsampling Bootstrapping *k*-fold validation Leave-one-out validation
**Significance Testing of ACE**	PACE (this paper) Boostrap confidence interval estimation (Efron and Tibshirani, 1997)

**Table 3:** List of methods applied to datasets. Each dataset was evaluated using PACE analysis with every possible combination of these methods. MAC = maximum allowed correlation.

Method	Options (Choice of one)
Peak Detection	On (Select only peaks) Off (Use the whole profile)
Feature Selection	Area under ROC curve (AUC) Fisher score J5 test Simple separability criterion t-test score Weighted separability criterion
De-correlation Enhancement	On (MAC < 1) Off (MAC = 1)
Classification Model	Naïve Bayesian Classifier Support Vector Machine (SVM)

**Figure A2:**
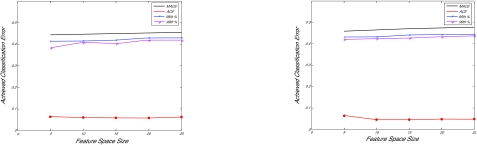
PACE analysis using a SVM-based classifier using the Fisher scoring criterion, with a 0.6 MAC threshold. Left Panel: performance without peak selection. Right panel: performance with peak selection.

**Figure A3:**
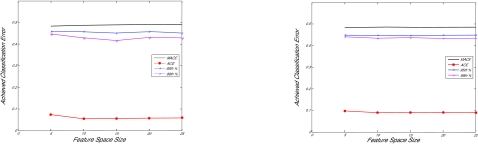
PACE analysis using a SVM-based classifier using the J5 scoring criterion, with a 0.6 MAC threshold. Left Panel: performance without peak selection. Right panel: performance with peak selection.

**Figure A4:**
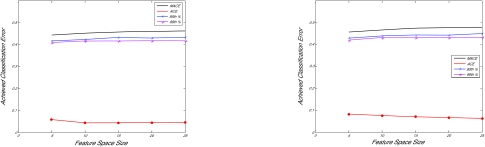
PACE analysis using a SVM-based classifier using the simple separability scoring criterion, with a 0.6 MAC threshold. Left Panel: performance without peak selection. Right panel: performance with peak selection.

**Figure A5:**
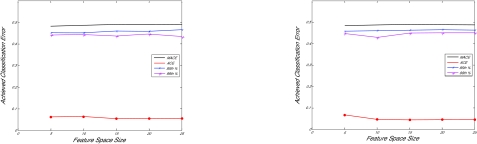
PACE analysis using a SVM-based classifier using the *t*-test scoring criterion, with a 0.6 MAC threshold. Left Panel: performance without peak selection. Right panel: performance with peak selection.

**Figure A6:**
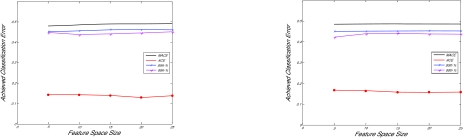
PACE analysis using a SVM-based classifier using the weighted separability scoring criterion, with a 0.6 MAC threshold. Left Panel: performance without peak selection. Right panel: performance with peak selection.

**Figure B2:**
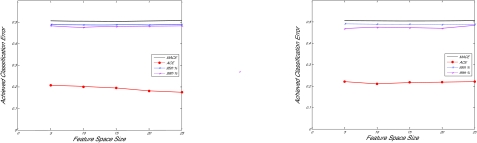
PACE analysis using a SVM-based classifier using the Fisher scoring criterion, with a 0.6 MAC threshold. Left Panel: performance without peak selection. Right panel: performance with peak selection.

**Figure B3:**
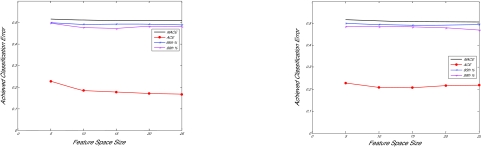
PACE analysis using a SVM-based classifier using the J5 scoring criterion, with a 0.6 MAC threshold. Left Panel: performance without peak selection. Right panel: performance with peak selection.

**Figure B4:**
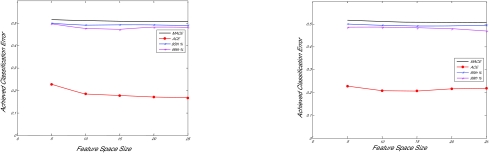
PACE analysis using a SVM-based classifier using the simple separability scoring criterion, with a 0.6 MAC threshold. Left Panel: performance without peak selection. Right panel: performance with peak selection.

**Figure B5:**
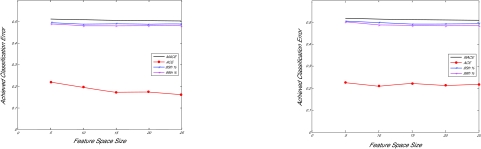
PACE analysis using a SVM-based classifier using the t-test scoring criterion, with a 0.6 MAC threshold. Left Panel: performance without peak selection. Right panel: performance with peak selection.

**Figure B6:**
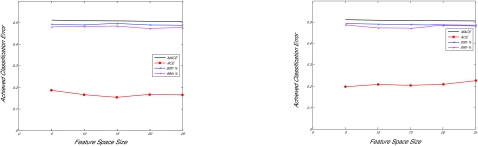
PACE analysis using a SVM-based classifier using the weighted separability scoring criterion, with a 0.6 MAC threshold. Left Panel: performance without peak selection. Right panel: performance with peak selection.

**Figure C2:**
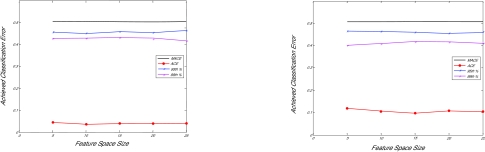
PACE analysis using a SVM-based classifier using the Fisher scoring criterion, with a 0.6 MAC threshold. Left Panel: performance without peak selection. Right panel: performance with peak selection.

**Figure C3:**
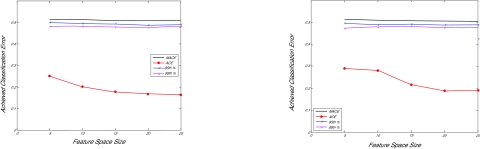
PACE analysis using a SVM-based classifier using the J5 scoring criterion, with a 0.6 MAC threshold. Left Panel: performance without peak selection. Right panel: performance with peak selection.

**Figure C4:**
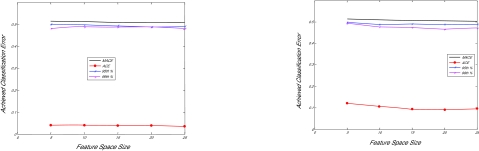
PACE analysis using a SVM-based classifier using the simple separability scoring criterion, with a 0.6 MAC threshold. Left Panel: performance without peak selection. Right panel: performance with peak selection.

**Figure C5:**
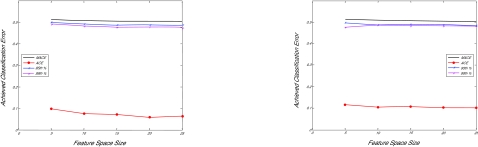
PACE analysis using a SVM-based classifier using the t-test scoring criterion, with a 0.6 MAC threshold. Left Panel: performance without peak selection. Right panel: performance with peak selection.

**Figure C6:**
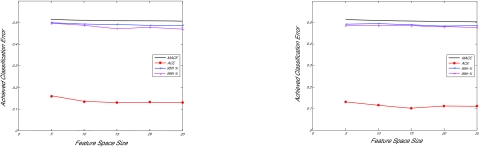
PACE analysis using a SVM-based classifier using the weighted separability scoring criterion, with a 0.6 MAC threshold. Left Panel: performance without peak selection. Right panel: performance with peak selection.

**Figure D2:**
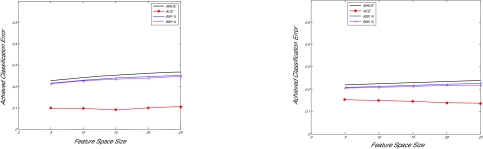
PACE analysis using a SVM-based classifier using the Fisher scoring criterion, with a 0.6 MAC threshold. Left Panel: performance without peak selection. Right panel: performance with peak selection.

**Figure D3:**
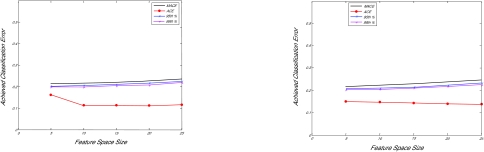
PACE analysis using a SVM-based classifier using the J5 scoring criterion, with a 0.6 MAC threshold. Left Panel: performance without peak selection. Right panel: performance with peak selection.

**Figure D4:**
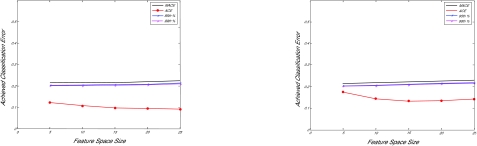
PACE analysis using a SVM-based classifier using the simple separability scoring criterion, with a 0.6 MAC threshold. Left Panel: performance without peak selection. Right panel: performance with peak selection.

**Figure D5:**
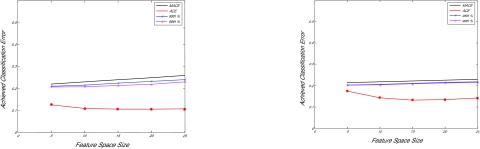
PACE analysis using a SVM-based classifier using the t-test scoring criterion, with a 0.6 MAC threshold. Left Panel: performance without peak selection. Right panel: performance with peak selection.

**Figure D6:**
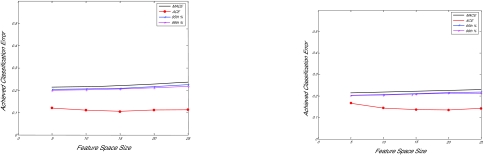
PACE analysis using a SVM-based classifier using the weighted separability scoring criterion, with a 0.6 MAC threshold. Left Panel: performance without peak selection. Right panel: performance with peak selection.
